# hCLE/RTRAF-HSPC117-DDX1-FAM98B: A New Cap-Binding Complex That Activates mRNA Translation

**DOI:** 10.3389/fphys.2019.00092

**Published:** 2019-02-18

**Authors:** Alejandra Pazo, Alicia Pérez-González, Juan Carlos Oliveros, Maite Huarte, Juan Pablo Chavez, Amelia Nieto

**Affiliations:** ^1^Centro Nacional de Biotecnología (CSIC), Madrid, Spain; ^2^CIBER de Enfermedades Respiratorias, Madrid, Spain; ^3^Department of Gene Therapy and Regulation of Gene Expression, Center for Applied Medical Research (CIMA), University of Navarra, Pamplona, Spain

**Keywords:** cap-binding, protein complexes, mRNA translation, translation activation, local translation

## Abstract

hCLE/C14orf166/RTRAF, DDX1, and HSPC117 are components of cytoplasmic mRNA-transporting granules kinesin-associated in dendrites. They have also been found in cytoplasmic ribosome-containing RNA granules that transport specific mRNAs halted for translation until specific neuronal signals renders them accessible to the translation machinery. hCLE associates to DDX1, HSPC117, and FAM98B in HEK293T cells and all four proteins bind to cap analog-containing resins. Competition and elution experiments indicate that binding of hCLE complex to cap resins is independent of eIF4E; the cap-binding factor needed for translation. Purified hCLE free of its associated proteins binds cap with low affinity suggesting that its interacting proteins modulate its cap association. hCLE silencing reduces hCLE accumulation and that of its interacting proteins and decreases mRNA translation. hCLE-associated RNAs have been isolated and sequenced; RNAs involved in mRNA translation are specifically associated. The data suggest that RNA granules may co-transport RNAs encoding proteins involved in specific functions together with RNAs that encode proteins needed for the translation of these specific RNAs and indicate an important role for hCLE modulating mRNA translation.

## Introduction

hCLE/C14orf166 also named RTRAF, is a 27 kDa protein with both nuclear and cytoplasmic localization (Huarte et al., 2001), which modulates different phases of RNA metabolism. In the nuclear compartment, it is a component of complexes involved in transcription-related functions such as the human spliceosome (Rappsilber et al., 2002), the 7SK snRNA methylphosphate capping complex (Jeronimo et al., 2007), and the tRNA-splicing ligase complex (Popow et al., 2011). hCLE has also an important role in the influenza virus life cycle, as it interacts with the viral polymerase (Huarte et al., 2001), positively modulates viral multiplication (Alfonso et al., 2011) and is incorporated into influenza virus particles (Rodriguez-Frandsen et al., 2016). In mature brain, hCLE is found in mRNA-containing kinesin-associated granules in dendrites (Kanai et al., 2004) and in developing brain, it has been found as a component of cytosolic, ribosome-containing RNA granules that transport specific mRNAs from the cell body to the dendrites, to allow local mRNA translation at sites distant from the nucleus (Elvira et al., 2006).

We recently described common hCLE interactors in the nucleus and cytosol by purification of hCLE complexes (Perez-Gonzalez et al., 2014). Purified hCLE complexes from both cell compartments contain DDX1, HSPC117 (Rtcb), and FAM98B, and endogenous hCLE fractionates in complexes with these proteins, with cytosolic complexes larger than nuclear complexes. We also showed that hCLE is a shuttling protein whose nuclear import requires active transcription, as do the import of DDX1 and HSPC117 proteins (Perez-Gonzalez et al., 2014). These observations suggest that, by forming a complex with DDX1, HSPC117, and FAM98B, hCLE shuttles between the nucleus and the cytoplasm to transport RNAs, and that this complex could be playing a prominent role in controlling RNA fate in the nucleus and in cytoplasm.

Most cellular mRNAs have a 5^′^ cap structure that prevents mRNA degradation and is essential for translation (Topisirovic et al., 2011). Eukaryotic translation initiation factor 4E (eIF4E) is the cap-binding component of the translation initiation eIF4F complex (Sonenberg, 2008; Topisirovic et al., 2011), which has a fundamental role on mRNA translation. The accessibility of eIF4E to the cap structure of mRNAs transported in neuronal RNA granules is crucial to allow accurate translation of specific neuronal RNAs at a precise site and time (Jiang and Schuman, 2002). Given (1) the presence of hCLE in cytoplasmic RNA-transporting granules involved in local translation in neurons, (2) its description as a component of nuclear and cytosolic protein complexes, and (3) its RNA-binding activity (Castello et al., 2012), it is a likely candidate for regulating mRNA transport or subsequent local mRNA translation. Here we studied whether hCLE binds cap structures, modulates translation and which cellular mRNAs are associated to purified hCLE. We show that hCLE complex has cap-binding activity and positively modulates mRNA translation.

## Materials and Methods

### Biological Materials

The human HEK293T cell line was provided by JC de la Torre (Scripps Research Institute; La Jolla, CA, United States). Plasmids pC-TAP, and pC-hCLE-TAP were described (Perez-Gonzalez et al., 2014). Lentiviral vectors for hCLE and control silencer expression were described (Perez-Gonzalez et al., 2014). Plasmid expressing the shRNA to silence eIF4E and the control plasmid that expresses a non-silencing shRNA derived from a transcript of the bacterium *Thermotoga maritima* have been previously reported (Burgui et al., 2007). pRSET-His-hCLE plasmid was cloned as described (Huarte et al., 2001). pCMV-Luc plasmid was kindly provided by I. Sola (CNB-CSIC). pGEM-T plasmids expressing N- and C-terminal hCLE parts were constructed by insertion of BclI fragments in pGEMT (Promega).

### Western Blotting

Western blotting was performed as described (Pérez-González et al., 2006), using as primary antibodies rabbit polyclonal anti-hCLE (Abcam, AB49342, 1:1000) and FAM98B (Abcam, AB179833, 1:500); mouse monoclonal anti-DDX-1 (Abcam, AB77213, 1:1000) and goat polyclonal anti-HSPC117/C22orf28 (LifeSpan BioSciences, LS-C139785, 1:000).

### Gel Silver Staining

Silver staining of glutaraldehyde-free gels suitable for proteomic approaches was performed as described (Perez-Gonzalez et al., 2014). Bands were visualized, excised, and analyzed by mass spectrometry.

### Protein Purification

Tandem affinity purification (TAP) was as described (Perez-Gonzalez et al., 2014). Poly-His-tagged hCLE was expressed in HEK293T cells by transfection of plasmids pRSET-His-hCLE in cells infected with a recombinant vaccinia virus expressing the phage T7 RNA polymerase (vTF7-3; provided by B. Moss, NIH, Bethesda MD) and purified as reported (Huarte et al., 2001).

### Cap Analog Binding

For binding to cap-analog resins, we used Sepharose 4B-7methyl GTP (GE-Healthcare 27-5025-01) and Sepharose 4B (Sigma 9012-36-6) as control. Protein extracts from HEK293T cells were diluted at least 1:10 in buffer (10 mM Tris-HCl pH 8, 100 mM KCl, 0.5 mM EDTA, 0.1% NP-40, 1 mM DTT, 1 mM PMSF) alone or in the presence of different competitors when indicated. After incubation (overnight, 4°C, with stirring), resin was washed with a buffer containing 10 mM Tris-HCl pH 8, 0.5 mM DTT, 0.2 mM EDTA, 100 mM NaCl, 0.5% Triton X-100 (washing buffer), laemmli buffer was added to the resin and the bound proteins analyzed by SDS–PAGE gel and Western blot.

For elution experiments, after binding of the extracts to the resin followed by extensive washes, increasing concentrations of different competitors were added sequentially to the same resin and after that laemmli buffer was added to the remaining resin. Eluted proteins and proteins remaining in the resin were analyzed in SDS–PAGE gels and Western blot. Input:bound protein ratios were: 1:12 in total and cytoplasmic HEK293T cell extracts; 1:6 in HEK293T cell nuclear extracts; 1:30 in His-tagged purified hCLE; 1:7.5 in competition experiments using His-hCLE purified protein.

### Characterization of hCLE- Associated RNAs

HEK293T cells were transfected with hCLE-TAP plasmid (pC-hCLE-TAP) or empty TAP (pC-TAP) as control. TAP purification was performed as described (Perez-Gonzalez et al., 2014) in RNA-preserving conditions to the tobacco etch virus protease (TEV) cleavage step. After TEV (Invitrogen) treatment, eluted proteins were proteinase-treated K (Sigma), followed by phenol-chloroform extraction and isopropanol precipitation. Precipitated RNAs were resuspended in DEPC-treated water and incubated with DNAse I (Ambion), followed by phenol-chloroform extraction to eliminate DNAse I and isopropanol-precipitated RNAs were resuspended as before. A similar number of hCLE-TAP- or TAP-expressing cells was used for high-throughput sequencing with TruSeq v3 chemistry and 50 bp single reads on an Illumina HiSeq 2000.

### RNA-Seq Analysis

Raw reads in FASTQ format were quality-checked with FASTQC^1^. For each sample, single-end reads were aligned against the human genome (primary_assembly, Ensembl release 84) with Bowtie2 (Langmead and Salzberg, 2012) with default parameters for single-end reads. Alignment files (BAM) were sorted and indexed with Samtools (Li et al., 2009) and visualized with IGV browser (Robinson et al., 2011).

Aligned reads were assigned to human genes with function htseq-count of the HTSeq package (Anders et al., 2015) with default parameters for single-end, strand-specific sequences (reverse), using full genes as features (Ensembl annotation version GRCh84.p5). Differential expression was estimated with bioconductor package DESeq2 (Love et al., 2014) with parameter cooks Cutoff = FALSE. *P*-values were adjusted by the FDR method (Benjamini et al., 2001).

### RNA-Seq Validation by Real Time Quantitative PCR

hCLE-CBD, and CBD associated RNAs from two different biological replicates were extracted as indicated before. Equal amounts of RNAs from each condition (adjusted to number of cells / volume) were used as template for cDNAs synthesis using the MultiScribe Reverse Transcriptase kit (Applied Biosystems), following supplier’s recommendations. 1/1, 1/10 and 1/100 dilutions of resulted cDNAs were used for Real Time Quantitative PCR using the Sybr Green system (Applied Biosystems). The following primers to amplify CHD6, Ribosomal 5S pseudogene 150, GAPDH and hCLE were used (forward; reverse): CHD6 [5^′^ CCAGGTAACCAAGGATATTG 3^′^; 5^′^CTCTCCTAGTCCACCTCTTT 3^′^]; Ribosomal S5 pseudogene 150 [5^′^ATCTCGTCTGATCTCGGAAGCTAAG 3^′^; 5^′^CGGTATTCCCAGGCGGTCTC 3^′^]; GAPDH [5^′^ GGAGTCAACGGATTTGGT 3^′^; 5^′^ GGTGGAATCATATTGGAACAT 3^′^]; hCLE [5^′^CAAACAAAAGAGGGCTTACCTG 3^′^; 5^′^ CTCCTCTATGTGCAGCAATC 3^′^]. Quantification was performed in a Real Time PCR System 7500 (Applied Biosystems) using the device’s software 7500 version 2.0.6. Quantification was performed using triplicates of each dilution.

### RNA Translation in hCLE-Silenced Cells

hCLE-silenced or control cells were prepared and selected as before (Perez-Gonzalez et al., 2014). Cells were transfected (0.5 μg/2 × 10^5^ cells) with the RNA encoding luciferase produced by *in vitro* transcription of a plasmid expressing firefly luciferase under the T3 promoter (Promega). Before transfection, the luciferase RNA was used for cap addition (Scriptcap m7G capping system, Cellscript) or was left uncapped. 1.5 h post-transfection cell extracts were prepared and used for luciferase detection by luminometric assay using luciferase reporter assay from Promega, measured in a Orion II, Microplate luminometer Titertek Berthold device, using Simplicity 4.2 program.

### Protein Radiolabeling Experiments

For radiolabeling experiments, HEK293T cells were infected with lentiviral vectors containing a hCLE silencer (sihCLE) or a control siRNA. Silenced cells were puromycin-selected as reported (Perez-Gonzalez et al., 2014). Cells were incubated with a Met/Cys-depleted DMEM (30 min), then left untreated, or treated with ActD (0.5 μg/mL) or with the RNAP III inhibitor (80 μM) in the same medium (30 min). Newly synthesized proteins were labeled with a ^35^S-Met-Cys mixture (50 μCi/mL) in the same medium and drug conditions. Labeled amino acids remained in the medium to the end of the experiment. Samples were collected at different times, quantitfication of total protein was evaluated by BCA assay (Thermo Scientific) and equal amounts of total protein analyzed by SDS–PAGE.

## Results

### Endogenous hCLE Complex Binds Cap Analog

The cap structure is an essential component of most mRNAs and it is crucial for translation through its interaction with the eIF4E factor (Sonenberg, 2008). The reported presence of hCLE in cytoplasmic RNA-transporting complexes involved in local translation in neurons, prompted us to test whether hCLE and its associated proteins had cap-binding activity.

**FIGURE 1:**
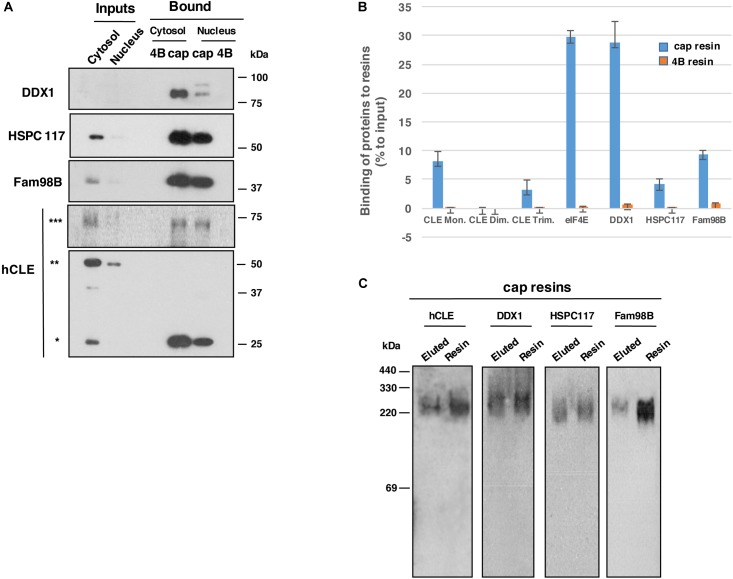
Endogenous hCLE complexes bind cap analog resins. **(A)** Nuclear and cytoplasmic extracts of HEK293T cells were incubated with control (4B) or cap analog (cap) resins. Western blot analysis of bound proteins using the corresponding antibodies are shown. **(B)** Graph showing the percentage of the indicated proteins bound to m^7^GTP analog resin (blue) or control resin (yellow), compared to the initial input that was incubated with the corresponding resins. Total extracts and the data of three independent experiments were used. **(C)** Total protein extracts from HEK293T cells were incubated with cap analog resin and eluted with 1 mM m^7^GTP. Eluted fractions and resin-bound proteins were resolved in non-denaturing gels and analyzed by Western blot with indicated antibodies. ^∗^monomer, ^∗∗^dimers, and ^∗∗∗^trimers. Cap-binding experiments were repeated more than five times. Input:bound protein ratios were: 1:12 in total and cytoplasmic HEK293T cell extracts; 1:6 in HEK293T cell nuclear extracts.

Nuclear and cytosolic fractions of HEK293T cells were used to evaluate binding of endogenous hCLE and of hCLE-interacting proteins to cap analog-containing resins. As reported, hCLE is present as a monomer and as denaturation-resistant dimers (Perez-Gonzalez et al., 2014). Gel filtration separation studies showed that in the nucleus, both mono- and dimeric hCLE comigrate with DDX1, HSPC117 and FAM98B, and RNAse treatment displaced hCLE monomers and dimers from DDX1-HSPC117-FAM98B, which remained together. In the cytosolic fraction, where hCLE is more abundant the hCLE monomer is associated to DDX1-HSPC117-FAM98B in untreated and RNAse-treated cells. In contrast, the hCLE dimer does not comigrate with the other three proteins (Perez-Gonzalez et al., 2014), which indicates that the dimer does not associate with these proteins in the cytoplasm. Cap-binding experiments using cell extracts that were not treated with RNAse were tested in SDS denaturing gels and showed that the monomer, but not the dimer, was retained in the cap analog resins in nuclear and cytosolic samples (Figure 1A), which suggests that hCLE-interacting proteins could participate in this activity. In these gels, the anti-hCLE antibody also recognized a band of ∼64 kDa in total extract, which was also retained by the cap analog resin. A band of this size was observed in previous hCLE-CBD purifications but was not analyzed. We excised this hCLE antibody-reactive band using silver staining and analyzed it by MALDI-TOF/TOF mass spectrometry system (Supplementary Table S1); we found that the band indeed corresponded to hCLE and should represent hCLE trimer.

The hCLE interactors DDX1, HSPC117, and FAM98B were also retained in the cap resin, as well as hCLE monomers and trimers, whereas they were excluded in control resin (Figure 1A). Quantitative data of the binding to cap-resins from three independent experiments were obtained. The data (Figure 1B) show the percentage of the components of hCLE complex and the cellular cap-binding factor eIF4E bound to m7GTP resins compared with control resins, using total cellular extracts from HEK293T cells.

To further characterize the hCLE complexes bound to cap structures, total extracts of HEK293T cells were applied to cap resin and eluted with 1 mM m^7^GTP (7-methylguanosine 5’-triphosphate); eluted and resin-bound fractions were resolved in non-denaturing gels as described (Niepmann and Zheng, 2006). hCLE, DDX1, HSPC117, and FAM98B were all present in 220–330 kDa complexes (Figure 1C), which indicates that hCLE complexes have cap-binding capacity.

### hCLE Forms Oligomers

**FIGURE 2:**
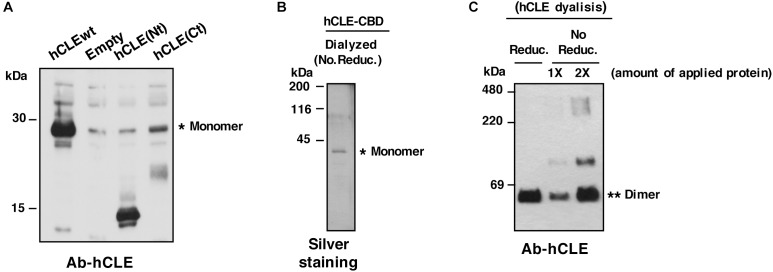
hCLE forms oligomers. **(A)** Total protein extracts of HEK293T cells transfected with plasmids expressing the whole hCLE sequence (hCLEwt), N-terminal domain [hCLE(Nt)] (aa 1–120) or C-terminal domain [hCLE(Ct)] (aa 120–244) were analyzed by Western blot with anti-hCLE antibodies. **(B)** Silver staining of hCLE-CBD purified protein dyalized in the presence of non-reducing agents (No. Reduc.). **(C)** Purified hCLE was dyalized in reducing (Reduc.) or non-reducing buffer (No Reduc.) and analyzed in non-denaturing gel followed by Western blot with anti-hCLE antibodies. ^∗^monomer and ^∗∗^dimers. 1X and 2X represent single or double amount of purified protein. Three independent experiments were done.

In addition to reported hCLE-hCLE dimers (Perez-Gonzalez et al., 2014), hCLE is thus able to form denaturation-resistant trimers. The most common cause of such resistant oligomers is the presence of coiled-coil regions, a structural motif in which heptad repeats containing hydrophobic residues fold into an α-helical secondary structure. In the water-filled environment of the cytoplasm, the most favorable arrangement of such helices is to wrap hydrophobic strands against each other, for exceptionally tight packing (Mason and Arndt, 2004). A coiled-coil domain is predicted in the hCLE sequence (Schou et al., 2014) and, using the ExPAsy COILS prediction program we found a strongly probable prediction for a coiled-coil domain at the hCLE C terminus (amino acids 194–233). HEK293T cells were transfected with plasmids encoding the amino- (Nt) or carboxy-terminal (Ct) hCLE domains (amino acids 1–120 and 120–244, respectively) and total cell extracts were used to analyze the migration of the expressed hCLE fragments using a polyclonal antibody that recognizes amino acids 85–134 of the protein (Abcam 49342). The results showed the migration predicted for the Nt (∼15 kDa), while the Ct migrated at 20–25 kDa, compatible with fragment dimerization (predicted size 12–14 kDa) (Figure 2A). These data suggest that the Ct region is responsible for hCLE oligomerization in denaturing conditions, possibly due to the presence of the coiled-coil domain.

Further characterization of hCLE oligomerization capacity was performed. Purified hCLE-CBD protein from cells transfected with TAP-hCLE was obtained as described (Perez-Gonzalez et al., 2014; Figure 2B), and dyalized in non-reducing condition (No Reduc.) or in a reducing buffer (Reduc.) (Figure 2B,C). The purified protein was analyzed in non-denaturing gels by silver staining (Figure 2B) or in non-denaturing gels followed by Western blot detection (Figure 2C). The protein dyalized in the absence of reducing agents showed the presence of multimeric forms, indicating the ability of purified hCLE to associate in multimers.

### Biochemical Properties of the Binding of hCLE Complex to Cap Analog

**FIGURE 3:**
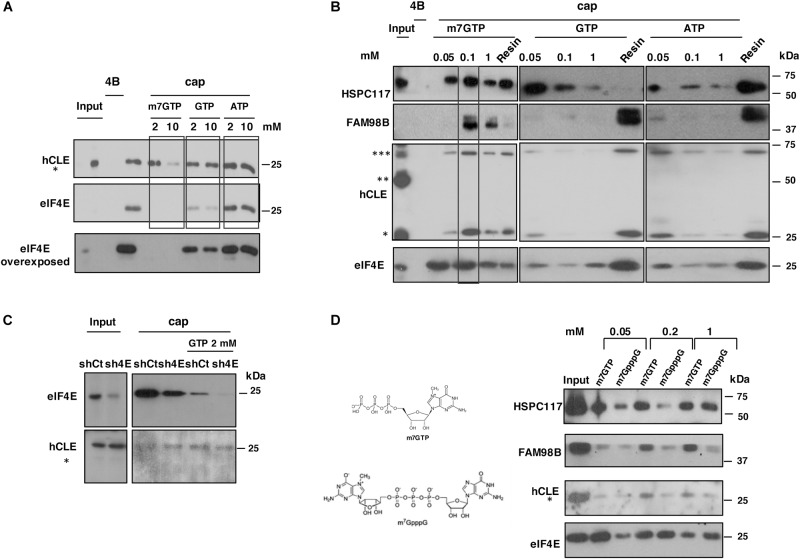
Biochemical properties of the hCLE-complex cap-binding activity. **(A)** Total extracts of HEK293T cells were incubated with cap analog (cap) or control resins (4B) in the presence of different concentrations of m^7^GTP, GTP or ATP (2 and 10 mM). Retained hCLE and eIF4E proteins in the corresponding resins after extensive washes were analyzed by Western blot. The experiments were repeated three times. **(B)** Total extracts of HEK293T cells were incubated with cap analog and control resins. After washing, protein was eluted sequentially with increasing concentrations of m^7^GTP, GTP or ATP (0.05, 0.1, and 1 mM). Eluted and resin-bound proteins were analyzed by Western blot. The experiments were repeated three times. **(C)** HEK293T cells were transfected with a control silencing plasmid (shCt) or with a plasmid specific for eIF4E silencing (sh4E). Total extracts were incubated with cap-analog resin alone or in the presence of GTP 2 μM and after washing, the retained proteins analyzed by SDS-gels and Western blots. **(D)** Total extracts of HEK293T cells were incubated with cap analog resins as indicated in part B. Protein was eluted sequentially with increasing concentrations of m7GTP, or m^7^GpppG (0.05, 0.2, and 1 mM). Eluted proteins were analyzed by Western blot and quantitated using ImageJ application (bottom). The experiments were repeated two times.

As factor eIF4E is the most relevant cap-binding protein modulating mRNA translation, we used several biochemical analyses to compare hCLE binding to cap analog with that of eIF4E. For competition experiments, we incubated HEK293T cell extracts with the cap analog-containing resin in the presence of various concentrations of m^7^GTP, GTP or ATP and the retained protein in the resins analyzed by SDS-denaturing gel followed by Western blot (Figure 3A). At 2 mM, m^7^GTP completely abolished eIF4E binding, whereas hCLE (analyzed as a monomer) remained resin-bound. Addition of 2 mM GTP also partially inhibited eIF4E binding to cap resin without affecting hCLE binding, whereas ATP had no effect on eIF4E or hCLE binding to cap analog resin.

Proteins bound to cap analog resins and eluted using different nucleotides were tested by adding competitor nucleotides to the resin, followed by re-elution with increasing concentrations of the same competitors to the same resin as described in “Materials and Methods.” Thus, the signals (Figure 3B) represent the proteins that were eluted sequentially through adding increasing concentrations of the competitor to the same resin and resin lane represents the remaining protein in the resin after the elution. The proteins were recovered and analyzed by SDS–PAGE (Figure 3B). A substantial amount of eIF4E was eluted by addition of 0.05 mM m^7^GTP to the cap resins; 0.1 mM m^7^GTP eluted more eIF4E, in agreement with its reported association constant of 0.119 mM for m7GTP (Zuberek et al., 2003). Addition of GTP or ATP only elute small amounts of eIF4E. For hCLE, addition of 0.1 mM m^7^GTP released the majority of the bound protein (monomer and trimer), whereas GTP or ATP had little effect. DDX1 (not shown), and FAM98B behaved similarly to hCLE in their cap resin binding. A portion of HSPC117 was eluted from the cap resins with low concentrations of GTP. HSPC117 is the essential subunit of a tRNA ligase complex that regulates tRNA maturation and hCLE is a component of the complex (Popow et al., 2011). The crystal structure of the HSPC117 protein from *Pyrococcus horikoshii* has been reported and showed that the crystalized protein coordinates Mn ion and binds GTP (Desai et al., 2013). Using a homology model of human HSPC117 in complex with manganese and covalently bound GMP built from the *P. horikoshii* crystal structure, it has been shown that the human protein contains a well-defined pocket for GTP binding (Nandy et al., 2017). These results indicate that HSPC117 alone has GTP binding activity and thus it is possible that a portion of the protein non-associated with hCLE-DDX1-FAM98B complex was eluted from cap-resins upon GTP addition and/or that GTP can dissociate a portion of HSPC117 from its binding partners. The next approach was the use of gene-silencing experiments using RNA interference. With this aim, HEK293T cells were transfected with a control silencing plasmid (shCt) or with a plasmid specific for eIF4E silencing (sh4E). As can be seen in Figure 3C, the eIF4E silencing plasmid efficiently decreased the accumulation levels of the eIF4E protein compared with the control plasmid, but did not modify the accumulation levels of hCLE. HEK293T cell extracts of control or eIF4E silenced cells were incubated with the cap analog-containing resin with or without the presence of 2 mM GTP and the proteins that remained resin-bound analyzed by SDS denaturing gels followed by Wester blots. According with previous results (Figure 3A), addition of 2 mM GTP inhibited eIF4E binding and did not substantially modify hCLE binding, analyzed as monomer.

Previous reports indicated that mononucleotide cap analogs interact more strongly with eukaryotic eIF4E than their dinucleotide counterparts (Ziemniak et al., 2015). Now we performed elution experiments as described above adding as competitor mononucleotide (m^7^GTP) or dinucleotide (m^7^GpppG) cap analogs at different concentrations. The eluted proteins were recovered and analyzed by SDS–PAGE (Figure 3D). As reported above, the addition of 50 μ m^7^GTP eluted an important fraction of eIF4E and HSPC117 and very little amount of FAM98B and hCLE (measured as monomer) and the addition of 50 μM m^7^GpppG did not elute important amounts of any of the tested proteins. All the proteins were efficiently eluted by addition of 200 μM or 1 mM m^7^GTP, but at these concentrations m^7^GpppG was capable to elute eIF4E, but almost nothing of hCLE, and its associated proteins, with the exception of HSPC117 that was efficiently eluted at 1 mM m^7^GpppG.

eIF4E and hCLE binding to cap-analogs resins show differences, such as competition by GTP for eIF4E but not for hCLE (Figure 3A,C), as well as differences in the elution experiments, where lower amounts of m^7^GTP are capable to elute bound eIF4E compared with hCLE (Figure 3B) and elution by m^7^GpppG of eIF4E (Figure 3D). Together the competition and elution experiments suggest that hCLE binding to cap resins is independent of eIF4E.

### Purified hCLE Binds Cap Analog

**FIGURE 4:**
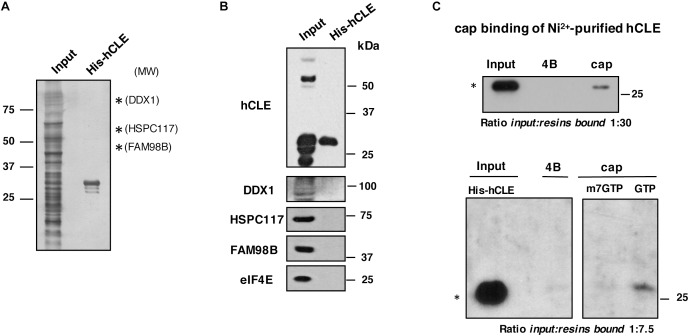
Purified hCLE binds cap analog. **(A)** Silver staining of Ni^2+^-purified His-hCLE protein. Only hCLE is visible after purification; arrows indicate molecular weight for associated proteins. **(B)** Western blot analysis of indicated proteins in total extracts (Input) or purified fraction of His-hCLE. To obtain the most accurate results, we analyzed all the proteins from the same sample and experiment. **(C)** (Top); Binding of Ni^2+^-purified His-hCLE proteins to cap analog and control resins. Bound protein was analyzed by Western blot. The relative amounts applied to SDS-gels were Input:resins bound 1:30. (Bottom); Purified His-hCLE was incubated with cap analog (cap) or control resins (4B) in the presence of 10 mM of GTP, or m^7^GTP as competitors and His-hCLE binding was analyzed by Western blot. The relative amounts applied to SDS-gels were Input:resins bound 1:7.5. The experiments were repeated at least three times. ^∗^monomer.

As hCLE and its associated proteins DDX1, HSPC117 and FAM98B had cap-binding activity, we tested whether hCLE alone has this activity. We expressed hCLE as a His-tagged protein at the N terminus (His-hCLE) in HEK293T cells and purified the protein by affinity Ni^2+^ chromatography, which allows stringent purification conditions; a representative silver staining of purified His-hCLE is shown (Figure 4A). Purified His-tagged hCLE protein was virtually free of its associated proteins (Figure 4B) as well as of the endogenous cap-binding protein, eIF4E. Cap-binding assays of the purified proteins (analyzed as a monomer) indicated that His-hCLE free of binding partners retained cap-binding activity (Figure 4C, top). Since previous data indicated that binding of endogenous hCLE to cap analog-containing resins is abolished by incubation with m^7^GTP, but not by GTP (Figure 3A), we performed cap-binding experiments of the purified His-hCLE in the presence of 10 mM m^7^GTP or GTP. Purified His-hCLE was retained in the cap-analog resins in the presence of 10 mM GTP, but its binding was abolished when the incubation was performed in the presence of 10 mM m^7^GTP (Figure 4C, bottom), in agreement with the behavior of the endogenous hCLE. These results indicated that His-hCLE free of binding partners retains cap-binding activity.

To examine whether the other components of the complex have cap-binding activity, HSPC117 and DDX1 were expressed as His-tagged proteins in HEK293T cells, followed by affinity Ni^2+^ chromatography purification, but both proteins were totally aggregated impeding their purification, therefore we cannot exclude additional cap-binding activity of other members of the hCLE complex. On the other hand, purified His-hCLE expressed by bacteria did not bind cap resin, suggesting that post-translational modifications of hCLE modulate its cap-binding activity. In addition, the His tag of hCLE or improper folding of the protein in the bacteria system can abolish the cap-binding. Remarkably, endogenous hCLE binds the cap resin better than purified His-hCLE where around 1% of the protein would be associated [input:bound protein ratios 1:12 in total and cytoplasmic HEK293T cell extracts (Figure 1, 3), compared with 1:30 in His-tagged purified hCLE], which suggest that hCLE complex components cooperate with hCLE to bind to cap structures. These results sustain the notion that hCLE belongs to the group of proteins with cap-binding capacity.

### hCLE Controls mRNA Translation

**FIGURE 5:**
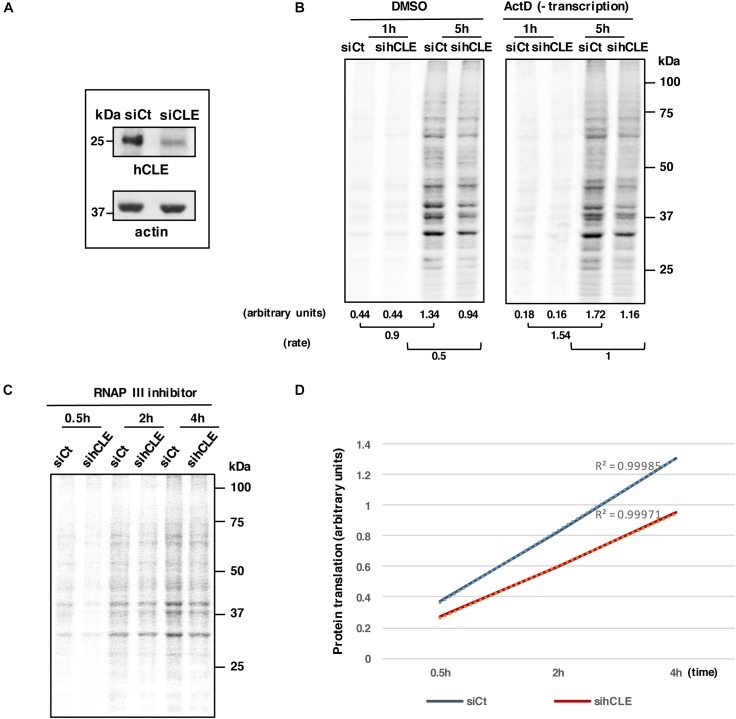
hCLE controls RNA translation. HEK293T cells were infected with lentivirus expressing a control siRNA (siCt) or a siRNA that targets hCLE (siCLE). **(A)** Western blot detection of hCLE in control or silenced cells. **(B)** HEK293T cells were infected with control (siCt) or a siRNA that targets hCLE lentiviruses. Control or silenced HEK293T cells were cultured in Met/Cys-depleted DMEM (30 min); cells were untreated (DMSO) or actinomycin D (Act D)-treated (30 min), followed by ^35^S-Met-Cys addition (time 0). At the indicated times aliquots were taken and used for SDS-gels and quantitated using ImageJ application (bottom). Rate represents the differences between signals obtained at 1h after ^35^S-Met-Cys addition and those obtained at 5 h. A representative image is shown. The experiment was repeated three times. **(C)** HEK293T cells were infected with lentivirus expressing a control siRNA (siCt) or a siRNA that targets hCLE. Control or silenced HEK293T cells were cultured in Met/Cys-depleted DMEM (30 min) and then treated with 80 μM of RNAP III inhibitor followed by ^35^S-Met-Cys addition (time 0). At the indicated times aliquots were taken and used for SDS-gels and quantitated using ImageJ application. A representative image is shown. **(D)** Quantitation of data obtained from part C. The experiment was repeated two times.

In addition to the reported involvement of hCLE in local translation in neurons, we have shown the ability of hCLE to bind cap structures. To analyze its possible role controlling mRNA translation, we established the translation rate of control and hCLE-silenced cells infected with lentiviruses that expressed control siRNA (siCt) or a hCLE-targeting siRNA (siCLE), respectively (Perez-Gonzalez et al., 2014; Figure 5A). The effect of hCLE silencing on the accumulation levels of the components of the complex taking from previous report, is shown in Supplementary Figure S1A.

Downregulation of hCLE inhibits RNAP II-directed transcription, thus to examine the effect of hCLE on the translation rate of accumulated mRNAs, we used siCt or siCLE cells, vehicle-treated (DMSO; dimethyl sulfoxide) or treated with actinomycin D (ActD) to inhibit RNA transcription and thus exclude hCLE effects on transcription (Pérez-González et al., 2006). A reduction of newly synthesized proteins measured by ^35^S-Met-Cys incorporation in hCLE silenced cell untreated or treated with actinomycin D was observed (Figure 5B). Similar results were obtained using a different RNAP II inhibitor such as 5,6-dichloro-1-beta-D-ribofuranosylbenzimidazole (DRB; data not shown). To discard possible effects of hCLE silencing on RNA stability we performed two different approaches. First, we transfected siCt or siCLE cells with a plasmid expressing pCMV-Luc (3 μg/1.2 × 10^6^) for 16 h, and then added DMSO or DRB to inhibit RNA transcription. Cell aliquots were collected at various times after treatment, cytoplasm and nucleus were separated, total cytoplasmic RNA was extracted and quantitated by nano-drop and luciferase mRNA was analyzed by Northern blot. Quantitation of luciferase RNA levels normalized by total RNA in control and silenced cells did not show variations (Supplementary Figure S1B), indicating that down-regulation of hCLE does not induce degradation of a exogenous RNA. Second, equal amount of hCLE silenced (siCLE) and control (siCTRL) HEK293T cells were used for separation of nuclear (Nuc.) and cytoplasmic (Cyt.) RNA. These RNAs were used for Dot-blot assay as described (Coloma et al., 2009) using poly-thimidine probes labeled with ^32^P. Nuclear and cytoplasmic RNAs were quantified in a phosphorimager device. Obtained quantification of absolute numbers is shown in Supplementary Figure S2 (arbitrary units). Results show that hCLE silencing does not affect the absolute quantity of messenger RNAs in the cells and thus, does not affect mRNA stability.

#### Effect of hCLE Complex Down-Regulation in Cells Treated With RNAP III Inhibitor

Down-regulation of hCLE provokes reduction not only of hCLE accumulation, but also of DDX1, HSPC117 and FAM98B proteins (Perez-Gonzalez et al., 2014). Since HSPC117 is the essential element of the tRNA-ligase complex (Popow et al., 2011), the reduced translation observed in hCLE silenced cells could be the consequence of a decreased accumulation of mature tRNAs. To discriminate between a general effect on RNA translation elicited by impaired tRNA maturation or by other effects, we performed mRNA translation experiments in the presence of an RNA polymerase III inhibitor (ML-60218, Calbiochem) in control and hCLE-downregulated cells. This compound is cell-permeable, has a IC50 = 27 μM for human RNAP III, and prevents RNAP III-mediated tRNA transcription. Previously, we checked the toxicity of the compound on a cell viability MTT assay (Levitz and Diamond, 1985). Total extracts of HEK293T cells treated with increasing concentrations of the RNAP III inhibitor (from 40–160 μM) were collected at various times post-treatment and MTT assays were performed. Addition of 40 and 60 μM drug during 5h did not affect cell viability, 80 μM produced a reduction of less than 20% in cell number and 160 μM was toxic for the cells, decreasing their viability more than 20%. Next, we monitored the effect of the RNAP III inhibitor on mRNA translation. For that HEK293T cells were transfected with the plasmid expressing pCMV-Luc (3 μg/1.2 × 10^6^) for 16 h, after that the cells were treated with DMSO or different concentrations of the inhibitor during 5 h, collected and processed for luciferase activity. A reduction on luciferase activity was observed in parallel with the addition of increasing concentrations of the drug (Supplementary Table S2). A reduction of around 35% was observed in cells treated with 80 μM of the RNAP III inhibitor; this concentration was then used for subsequent experiments.

To examine the effect of hCLE down regulation on RNA translation in conditions of RNAP III inhibition, control or down-regulated hCLE cells were treated with 80 μM RNAP III inhibitor during 5 h. After that, newly synthesized proteins were measured at different times after addition of ^35^S-Met-Cys through autoradiography and quantitation of the samples applied to SDS-gels (Figure 5C,D). Decreased incorporation of label was observed in the hCLE silenced cells compared with control cells.

#### Effect of hCLE Complex Down Regulation on Cap-Dependent mRNA Translation

To further characterize the ability of hCLE to modulate mRNA translation in a manner independent of its effect on tRNAs maturation, additional experiments were performed. Control and hCLE-silenced cells were transfected with *in vitro* transcribed luciferase RNA that was used for cap addition or was left uncapped after *in vitro* transcription as described in Methods section. The corresponding uncapped or capped luciferase RNAs were used for transfection and at different hours post-transfection cell extracts were prepared and luciferase activity monitored. The results are shown in Supplementary Table S3. A small decrease in luciferase activity was found in hCLE down-regulated cells transfected with uncapped luciferase RNA, probably as the consequence of hCLE on tRNA maturation, while the translation of capped luciferase RNA was clearly decreased in hCLE-silenced cells. Similar results were obtained using 1.5 or 3 h post-transfection (hpt); to minimize possible degradation effects 1.5 hpt. was used in subsequent assays.

**FIGURE 6:**
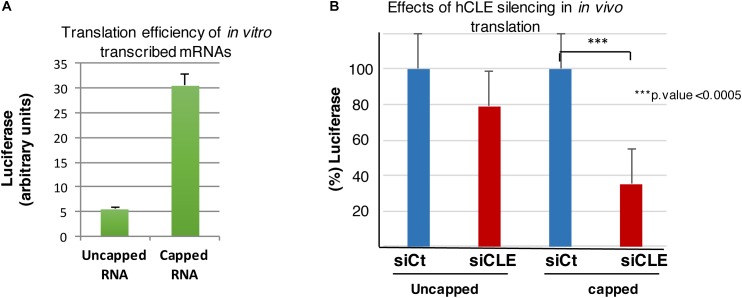
hCLE controls cap-dependent translation. **(A)** *In vitro* transcribed luciferase RNA was left uncapped or capped and both RNAs used for luciferase determination by *in vitro* translation assay. **(B)** HEK293T cells were transfected with 0.5 μg/2 × 10^5^ cells of *in vitro* transcribed luciferase RNA uncapped or capped and 1.5 h post-transfection luciferase activity was analyzed. The graphic shows the percentage of translated uncapped or capped luciferase RNA in control (taking as 100%) and hCLE silenced cells. Four independent experiments with three technical replicates were done. ^∗∗∗^*p*-value < 0.0005.

In the following experiments, the efficiency of cap addition on the RNA used for transfection was previously monitored by *in vitro* translation of capped luciferase RNA compared with uncapped RNA (Figure 6A). The results showed an increase in *in vitro* translation of the capped luciferase RNA of 5.5 folds, indicating an efficient cap addition. The luciferase activity in control or hCLE silenced cells after 1.5 h post-transfection from three independent experiments is shown in Figure 6B. Again, a small decrease on luciferase activity was found in hCLE down-regulated cells transfected with uncapped luciferase RNA; this decrease may be the consequence of reduced levels of mature tRNAs. In contrast, the translation of capped luciferase RNA was highly decreased in hCLE-silenced cells (Figure 6B).

Together our data support the notion that depletion of the hCLE complex decreases mRNA translation in addition to its role reducing production of mature tRNAs and suggest that hCLE probably in complex with DDX1-HSPC117-FAM98B, positively modulates regular cap-dependent mRNA translation.

#### hCLE Binds Specific mRNAs

**FIGURE 7:**
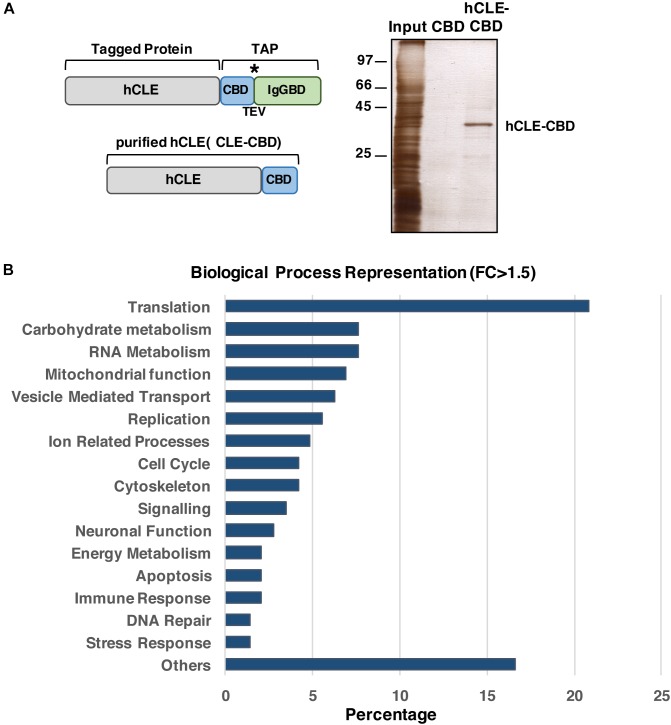
hCLE binds specific messenger RNAs. **(A)** Scheme of the hCLE-TAP fusion protein used for tandem affinity purification (TAP) and the final purified protein hCLE-CBD. Peptides bearing the calmodulin-binding domain (CBD) and the IgG-binding domain of *Staphylococcus aureus* protein A (IgGBD) are separated by a cysteine protease (TEV) cleavage sequence. **(B)** Silver staining of hCLE-CBD purified protein used for high throughout sequencing. **(C)** Percent representation of mRNAs associated to hCLE-CBD, grouped by biological function according to GeneOntology annotations. Two biological replicates were used. ^∗^represents the TEV protease cleavage site.

Previous reports indicated that hCLE is an RNA-binding protein (Castello et al., 2012). To identify the mRNAs specifically bound by hCLE, we expressed a carboxy-terminal hCLE-TAP (tandem affinity purification) tagged protein or control TAP proteins in HEK293T cells and purified these proteins as described (Perez-Gonzalez et al., 2014), followed by extraction of bound RNAs. The TAP tag contains an IgG-binding domain from protein A (IgGBD) and a calmodulin-binding domain (CBD) separated by a TEV protease cleavage site (see a scheme on Figure 7A). The starting and final hCLE proteins and silver staining of the affinity-purified hCLE-CBD are shown (Figure 7B). We used RNAs isolated from two independent purification replicates using TAP- and hCLE-TAP-expressing cells (which produced CBD and hCLE-CBD, respectively) for high-throughput sequencing. Using >1.5-fold change and *p*-adjustment < 0.05 as cut-off parameters, we found 144 RNAs bound specifically to hCLE-CBD compared with CBD (GEO accession number: GSE84824) (Supplementary Table S4). The biological processes in which these hCLE-bound RNAs are involved are shown in Figure 7C. RNA-encoding proteins involved in mRNA translation are clearly enriched. In addition, there are RNAs encoding proteins that control carbohydrate metabolism and mitochondrial function; these proteins can provide the necessary energy for mRNA translation since it has a huge energy demand. Interestingly, RNAs involved in neuronal activity such as neurite growth-promoting factor 2 (Winkler and Yao, 2014) or sigma-1 receptor (Takebayashi et al., 2004) were found associated to hCLE. The presence of mRNAs with neuronal function in HEK293 cells is not surprising since transcriptome analysis of these cells revealed a high proportion of mRNAs normally expressed specifically in neuronal cells^2^ (Shaw et al., 2002; Lin et al., 2014), giving HEK293 a phenotype surprisingly similar to neuronal lineage cells such as PC12 or Ntera-2. The RNA-seq results were confirmed by quantitative RT-PCR analysis of two RNAs non-associated or associated with purified hCLE (Supplementary Figure S3).

The population of mRNAs bound to hCLE is not very high, suggesting the presence of specific features in the RNAs associated that would be recognized by hCLE. Therefore, we analyze the occurrence of common motifs within the bound RNAs performing a bioinformatic study at both the 5^′^ and the 3^′^ UTR of the associated mRNAs^3^^,^^4^ (Supplementary Tables S5, S6, respectively). Several enriched common motifs were found both at the 5^′^ and 3^′^ ends of the bound RNAs. These data suggest that hCLE could recognize specific motifs in the associated RNAs; it should be taken into account the possibility that these motifs can work individually or cooperatively or even with other factors such as structural features to draw the selection of mRNAs recognized by hCLE.

## Discussion

Most cellular mRNAs have a 5^′^ cap structure that prevents mRNA degradation and is essential for translation (Topisirovic et al., 2011). Two main eukaryotic cap-binding proteins have been identified to date; they include nuclear cap-binding complex (nCBC), comprised of a cap-binding subunit (CBP20) and an auxiliary protein (CBP80), and eukaryotic translation initiation factor 4E (eIF4E) in cytoplasm, which is a component of the translation initiation eIF4F complex (Sonenberg, 2008; Topisirovic et al., 2011). All eukaryotes express multiple eIF4E family members (Rhoads, 2009) and metazoan contain a homolog of eIF4E named 4EHP, that has lower affinity for cap analogs and cap structure than eIF4E (Zuberek et al., 2007). Binding of eIF4E to cap structure is positively regulated by the eIF4G component of the eIF4F complex and by the eIF4E-binding proteins (Sonenberg, 2008) and negatively regulated by 4E transporter (Sonenberg and Hinnebusch, 2009). The 5^′^ cap has a prominent role in mRNA metabolism, since it prevents RNA degradation by 5’ exonucleases (Evdokimova et al., 2001). Undesirable mRNAs are stored in P-bodies for temporary storage or decapping (Parker and Sheth, 2007) and the decapping complex must compete with eIF4E for cap structure; thus the 5^′^ cap structure is a marker of actively translating mRNAs and regulates mRNA half-lives (Sonenberg and Gingras, 1998).

Recently a new cellular translation pathway has been described that relies on a previously unknown cap-binding activity of eIF3d, a subunit of the eukaryotic initiation factor 3 complex, which is utilized by a subset of mRNAs required for cell proliferation (Lee et al., 2016). Additional cap-binding proteins have been reported such as IFIT1 that recognizes the 5^′^ end of viral mRNAs and can compete with eIF4F to selectively bind and sequester viral cap-mRNA (Kumar et al., 2014). Other cap-binding proteins such as LARP1 seem to be implicated in the selective translational regulation of mRNAs with terminal oligopyrimidine tracts (Tcherkezian et al., 2014). Hence, in addition to the general pathway used for most of the cap-mRNAs for translation, there are specialized proteins required for translation of specific mRNAs.

Here, we describe a new cap-binding complex composed by hCLE-DDX1-HSP117-FAM98B proteins. hCLE/C14orf166 is a component of many different protein complexes, including the nuclear tRNA splicing ligase complex (Popow et al., 2011), the splicesome complex (Rappsilber et al., 2002), the 7SK snRNA methylphosphate capping complex (Jeronimo et al., 2007), and the heterogeneous nuclear and cytosolic ribonucleoprotein complex characterized through TDP-43 protein purification (Freibaum et al., 2010). hCLE is found in cytosolic RNA granules that transport mRNAs to axons and dendrites in neurons and are important for synaptic plasticity. In neuronal RNA-transporting kinesin-associated granules, hCLE and staufen-1 are among the “core” protein components (Kanai et al., 2004), accordingly, staufen-1 purification from HEK293T cells shows associated hCLE (Villacé et al., 2004). In developing brain, neuronal RNA granules involved in local mRNA translation also contain hCLE as well as ribosomes and the motor protein dynein (Elvira et al., 2006). Proteomic studies in neurons identified a number of DEAD box proteins as components of RNA granules and hCLE was detected using DEAD box 1 (DDX1) as a marker (Miller et al., 2009).

This heterogeneous repertoire of hCLE-containing complexes suggests their dynamic nature, and indicates that distinct functional complexes may share some proteins and have others that are specific. The association-dissociation of certain proteins in these complexes might be the force that modulates their function. hCLE is closely associated with DDX1, HSPC117, and/or FAM98B not only in HEK293T cells (Perez-Gonzalez et al., 2014), but in complexes such as the tRNA splicing ligase complex (with DDX1, HSPC117, and FAM98B), the splicesome complex (with DDX1), the staufen complex (with DDX1), kinesin granules (with DDX1, HSPC117), dynein granules (with DDX1, HSPC117), the DDX1 complex (with DDX1) or the TDP-43 complex (with DDX1, FAM98B). hCLE-DDX1-HSPC117-FAM98B can therefore be considered a functional complex, and depletion of HSPC117 (Popow et al., 2011) or hCLE (Perez-Gonzalez et al., 2014) provokes downregulation of the other three complex components.

Here, we show that in association with its partners, hCLE binds cap analog-containing resins in HEK293T cells (Figure 1, 3) and purified hCLE virtually free of other proteins maintains cap-binding activity (Figure 4), which is nonetheless clearly reduced, which suggests that the hCLE-DDX1-HSPC117-FAM98B proteins form the functional cap-binding complex. The data suggest that hCLE complex binds cap structures independently of eIF4E, the conventional cytosolic cap-binding factor (Figure 3). The different behavior of hCLE complex and eIF4E protein in cap-binding assays could be particularly relevant in neurons. In dendrites, the translation of RNP-transported RNAs must be prevented until specific neuronal signals provoke RNP rearrangement and make the RNAs accessible to the translation machinery. A role of hCLE complex modulating the translation of specific mRNAs in response to particular signals might operate in local neuronal mRNA translation.

## Author Contributions

AP, AP-G, and AN designed the research studies. AP and AP-G conducted the experiments. AP, JO, and JCh carried out the bioinformatics analyses and analyzed the data, MH made plasmids construction and expression. AN wrote the manuscript.

## Conflict of Interest Statement

The authors declare that the research was conducted in the absence of any commercial or financial relationships that could be construed as a potential conflict of interest.

## Footnotes

1 http://www.bioinformatics.babraham.ac.uk/projects/fastqc

2 https://www.novoprolabs.com/support/articles/a-hek293-cell-database-201607181270.html

3 http://bioinfogp.cnb.csic.es/files/users/anieto/TAP_CLE_UTR_analysis/out_findMotif_All_5UTR/homerResults.html

4 http://bioinfogp.cnb.csic.es/files/users/anieto/TAP_CLE_UTR_analysis/out_findMotif_All_3UTR/homerResults.html
