# Supplementary material for: hCLE/RTRAF-HSPC117-DDX1-FAM98B: A New Cap-Binding Complex That Activates mRNA Translation
Source: Front Physiol. 2019 Feb 18;10:92. doi: 10.3389/fphys.2019.00092 (PMC6388641; doi:10.3389/fphys.2019.00092)
Supplement: Supplementary file 1 [file Data_Sheet_1.PDF]

Supp. Fig.S1

A

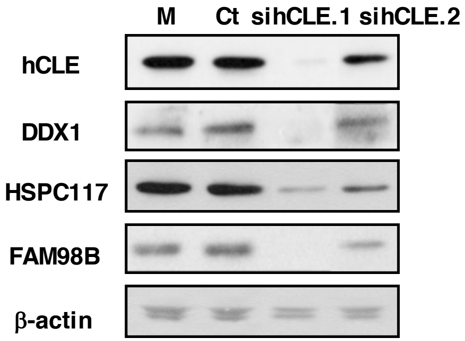

**FromPérez-Gonzalez et al, 2014*

B

Treatment	Sample	Luciferase RNA (arbitrary units)	Luc RNA/ Total RNA
DMSO	si.Ct 1h	72.8	9
	si.Ct 2,30 h	77.7	8
	si.Ct 5h	73.4	10.0
	si.CLE 1h	62.9	10.5
	si.CLE 2,30 h	77.7	8.2
	si.CLE 5h	71.8	11.2
DRB	si.Ct 1h	55.5	7.0
	si.Ct 2,30 h	60.8	6.5
	si.Ct 5h	59.7	7.0
	si.CLE 1h	53.0	7.9
	si.CLE 2,30 h	49.0	7.0
	si.CLE 5h	56.1	7.8

Supp. Fig. S1. Silencing of hCLE does not modify RNA stability. of an exogenous mRNA. A). Silencing of hCLE with different lentiviral silencers (siCLE.1 and siCLE.2) causes a reduction in the levels of hCLE and its associated-proteins; DDX1, HSPC117 and Fam98B, compared to the mock cells (M) and control silencer treated cells (Ct) (From Perez-Gonzalez et. al. 2014). B). siCt and siCLE HEK293T cells were transfected with plasmid pCMV-Luc expressing luciferase (16 h) and DMSO- or DRB-treated. Aliquots collected at various times were fractioned to separate cytoplasm from nucleus. Total RNA quantity was measured by nano-drop and Luc mRNA amount was analyzed and measured by Northern blot analysis.
