# Supplementary material for: hCLE/RTRAF-HSPC117-DDX1-FAM98B: A New Cap-Binding Complex That Activates mRNA Translation
Source: Front Physiol. 2019 Feb 18;10:92. doi: 10.3389/fphys.2019.00092 (PMC6388641; doi:10.3389/fphys.2019.00092)
Supplement: Supplementary file 2 [file Data_Sheet_2.PDF]

Supp. Fig.S2

	si.Ct		si.CLE	
	Cyt.	Nuc.	Cyt.	Nuc.
Sample 1	4.7	4.4	4.7	4.2
Sample 2	4.8	4.2	4.7	4.3
Sample 3	4.7	4.5	4.7	4.3

Supp. Fig. S2. Silencing of hCLE does not affect RNA stability. Equal amounts of control (si.Ct) or hCLE silenced (si.CLE) HEK293T cells were used for separation of nuclear (Nuc.) and cytoplasmic (Cyt.) RNA and used for dot-blot assay using Poly-thimidine probes labelled with ^{32}P. Nuclear and cytoplasmic RNAs were quantified in a phosphorimager device. Obtained quantification of absolute numbers is shown (arbitrary units).
