# Supplementary material for: hCLE/RTRAF-HSPC117-DDX1-FAM98B: A New Cap-Binding Complex That Activates mRNA Translation
Source: Front Physiol. 2019 Feb 18;10:92. doi: 10.3389/fphys.2019.00092 (PMC6388641; doi:10.3389/fphys.2019.00092)
Supplement: Supplementary file 3 [file Data_Sheet_3.PDF]

Supp. Fig. S3

A

	CHD6		Ribosomal 5S pseudogene		GAPDH		CLE	
	Mean	*STDEV*	*Mean*	*STDEV*	*Mean*	*STDEV*	*Mean*	*STDEV*
**CBD (Cts)**	26.983	0.007	18.704	0.046	11.893	0.027	24.151	0.058
**CLE-CBD (Cts)**	26.642	0.204	18.887	0.029	7,027	0.059	16.898	0.004
**Ct Difference**	0.341		-0.183		4.865		7.253	

B

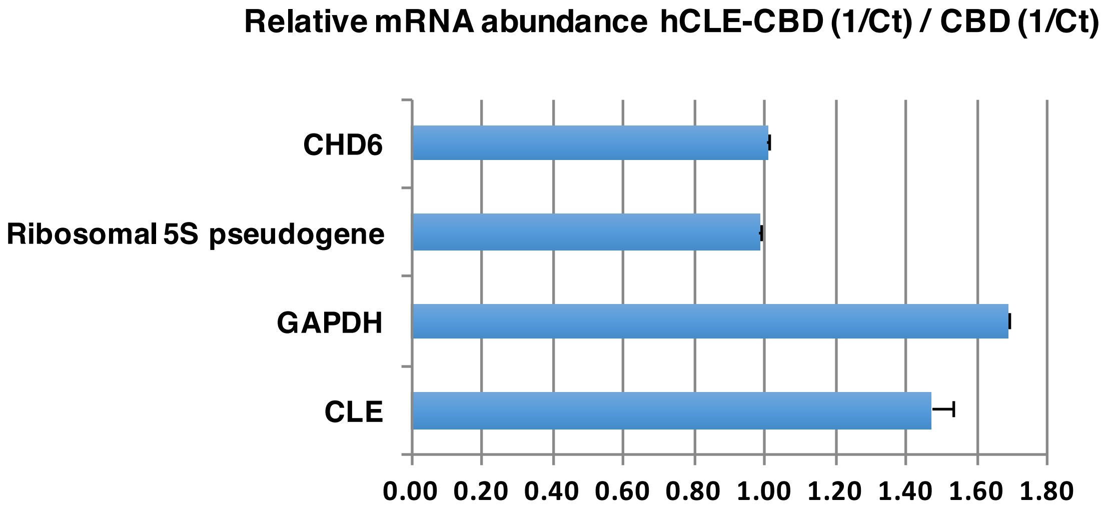

Supp. Fig. S3. Validation of RNAs bound to purified hCLE-CBD. RNAs bound to CBD or hCLE-CBD were used for qRT-PCR to detect the abundance of non-associated (CHD6 and Ribosomal 5S pseudogene) or associated (GAPDH and hCLE) mRNAs to purified CLE-CBD. A). Table showing the Ct amplification of the selected mRNAs in CBD and CLE-CBD RNAs. Mean and standard deviation of three independent measures is represented. B). Diagram showing relative abundance of each mRNAs taking as 1.00 the 1/Ct values found in the CBD sample.
