# Supplementary material for: hCLE/RTRAF-HSPC117-DDX1-FAM98B: A New Cap-Binding Complex That Activates mRNA Translation
Source: Front Physiol. 2019 Feb 18;10:92. doi: 10.3389/fphys.2019.00092 (PMC6388641; doi:10.3389/fphys.2019.00092)
Supplement: Supplementary file 4 [file Data_Sheet_4.PDF]

Supp Table S1. Identification of hCLE as monomer, dimer and trimer in denaturing gel conditions

	Protein name	Accesion Number	MW	Pept (MS(MSMS))	Score	Seq.Cov
64kDa	hCLE/c14orf166	gil55613379	28066	19(5)	207	74
48kDa	hCLE/c14orf166	gil55613379	28066	14(1)	150	45.4
27kDa	hCLE/c14orf166	gil55613379	28066	11	135	42.6

AC: Accession Number of the top protein from NCBInr protein Database (non-identical NCBI protein database).

MW (Mr): Nominal molecular weight of each protein.

Pept (MS[MSMS]): number of matched peptides from the top scoring protein in peptide mass fingerprinting and number of MS/MS spectra that were matched to this protein.

Score: Mascot protein score. This number reflects the combined scores of all observed mass spectra that can be matched to amino acid sequences within that protein. A higher score indicates a more confident match.

Seq Cov: Percentage of the database protein sequence covered by matching peptides

hCLE reactive bands from HEK293T cells visualized at different molecular weights (64kDa, 48kDa and 25 kDa) were cut and analyzed by MASCOT mass spectrometry.
