# Supplementary material for: hCLE/RTRAF-HSPC117-DDX1-FAM98B: A New Cap-Binding Complex That Activates mRNA Translation
Source: Front Physiol. 2019 Feb 18;10:92. doi: 10.3389/fphys.2019.00092 (PMC6388641; doi:10.3389/fphys.2019.00092)
Supplement: Supplementary file 5 [file Data_Sheet_5.PDF]

Supp Table S2. Effect of different concentrations of RNAP III inhibitor on luciferase activity

	Luciferase (Mean)	Luciferase (STDEV)	Percentage
DMSO	136272	48415	100
RNAP III In. 20 µM	113797	7901	83.5
RNAP III In. 40 µM	108003	41263	79.2
RNAP III In. 60 µM	103172	24315	75.7
RNAP III In. 80 µM	93141	16029	68.3

HEK293T cells were transfected with a plasmid expressing pCMV-Luc for 16 h, after that the cells were treated with DMSO or different concentrations of the RNAP III inhibitor during 5 h, collected and processed for luciferase detection by luciferase reporter assay from Promega .
