# Supplementary material for: hCLE/RTRAF-HSPC117-DDX1-FAM98B: A New Cap-Binding Complex That Activates mRNA Translation
Source: Front Physiol. 2019 Feb 18;10:92. doi: 10.3389/fphys.2019.00092 (PMC6388641; doi:10.3389/fphys.2019.00092)
Supplement: Supplementary file 6 [file Data_Sheet_6.PDF]

Supp Table S3. Effect of hCLE silencing on uncapped and capped luciferase-mRNA translation

luciferase activity

	1.5 hpt			3 hpt		
	siCLE	siCt	siCt/siCLE	siCLE	siCt	siCt/siCLE
**uncapped LUC-RNA**	728	1160	1.59	1163	2262	1.94
**capped LUC-RNA**	34812	133296	3.82	152302	521574	3.42

Control (siCt) or hCLE silenced (siCLE) HEK293T cells were transfected with equal amounts of in vitro transcribed luciferase RNA uncapped or capped. At the indicated h post-transfection luciferase activity was analyzed by luciferase reporter assay from Promega. The mean of two independent replicates is shown.
