# Supplementary material for: hCLE/RTRAF-HSPC117-DDX1-FAM98B: A New Cap-Binding Complex That Activates mRNA Translation
Source: Front Physiol. 2019 Feb 18;10:92. doi: 10.3389/fphys.2019.00092 (PMC6388641; doi:10.3389/fphys.2019.00092)
Supplement: Supplementary file 7 [file Data_Sheet_7.PDF]

Supp. Table S4. List of proteins encoded by the mRNAs bound to purified hCLE with fold change >1.5

ID	Gene Name	Decription	Fchange	Adj. P-Value	Gene Ontology Annotation
ENSG00000087302	C14orf166	chromosome 14 open reading frame 166	5.34	0.00000061	
ENSG00000281508	CDR1-AS	CDR1 antisense RNA [Source:HGNC	3.35	0	Progression of Alzheimer disease
ENSG00000111640	GAPDH	glyceraldehyde-3-phosphate dehydrogenase	2.67	0	Carbohidrate metabolism
ENSG00000089157	RPLP0	ribosomal protein, large, P0	2.56	0	Translation
ENSG00000169100	SLC25A6	solute carrier family 25 (mitochondrial carrier; adenine nucleotide translocator)	2.56	0	Protein carrier to mitochondria
ENSG00000172500	FIBP	fibroblast growth factor (acidic) intracellular binding protein	2.48	0.00843095	Fibroblast growth factor receptor signalling pathway
ENSG00000140988	RPS2	ribosomal protein S2	2.44	0	Translation
ENSG00000169692	AGPAT2	1-acylglycerol-3-phosphate O-acyltransferase 2	2.44	0.00880524	Phospholipid metabolic process
ENSG00000087086	FTL	ferritin, light polypeptide	2.38	0.00000001	Iron ion transport
ENSG00000112514	CUTA	cutA divalent cation tolerance homolog (E. coli)	2.33	0.00012812	Response to metal ion
ENSG00000125534	PPDPF	pancreatic progenitor cell differentiation and proliferation factor	2.32	0.00058846	Cell differentiation
ENSG00000116521	SCAMP3	secretory carrier membrane protein 3	2.29	0.00262678	Vesicle mediated transport
ENSG00000130313	PGLS	6-phosphogluconolactonase	2.29	0.00568708	Carbohydrate metabolism
ENSG00000147955	SIGMAR1	sigma non-opioid intracellular receptor 1	2.28	0.00000092	Nervous system development
ENSG00000122140	MRPS2	mitochondrial ribosomal protein S2	2.26	0.0009083	Mitochondrial function
ENSG00000079462	PAFAH1B3	platelet activating factor acetylhydrolase 1b catalytic subunit 3	2.23	0.03199731	Lipid metabolic proteins. Brain development
ENSG00000167513	CDT1	chromatin licensing and DNA replication factor 1	2.19	0.00002072	DNA replication checkpoint
ENSG00000005448	WDR54	WD repeat domain 54	2.18	0.03327398	Protein binding
ENSG00000089327	FXYD5	FXYD domain containing ion transport regulator 5	2.18	0.00981658	Ion transport
ENSG00000108479	GALK1	galactokinase 1	2.16	0.03888922	Carbohydrate metabolic process
ENSG00000137309	HMGA1	high mobility group AT-hook 1	2.16	0.00021528	Transcription
ENSG00000074071	MRPS34	mitochondrial ribosomal protein S34	2.15	0.00002857	Mitochondrial function
ENSG00000007080	CCDC124	coiled-coil domain containing 124	2.15	0.00182167	Cell cycle
ENSG00000103202	NME4	NME/NM23 nucleoside diphosphate kinase 4	2.15	0.00000394	Nucleotide metabolism
ENSG00000167645	YIF1B	Yip1 interacting factor homolog B (S. cerevisiae)	2.15	0.0235604	Uncharacterized
ENSG00000137404	NRM	nurim (nuclear envelope membrane protein)	2.15	0.02445496	Nuclear envelop protein
ENSG00000204628	GNB2L1	guanine nucleotide binding protein (G protein), beta polypeptide 2-like 1	2.14	0	Cell cycle
ENSG00000161179	YDJC	YdjC homolog (bacterial)	2.12	0.00535578	Uncharacterized
ENSG00000110492	MDK	midkine (neurite growth-promoting factor 2)	2.07	0.00023882	Nervous system development
ENSG00000130811	EIF3G	eukaryotic translation initiation factor 3 subunit G	2.07	0.00090737	Translation
ENSG00000065978	YBX1	Y-box binding protein 1	2.05	0.00001362	Transcription
ENSG00000149925	ALDOA	aldolase, fructose-bisphosphate A	2.05	0	Carbohydrate metabolic process
ENSG00000013306	SLC25A39	solute carrier family 25 member 39	2.04	0.00491229	Translation
ENSG00000137106	GRHPR	glyoxylate reductase/hydroxypyruvate reductase	2.04	0.00544704	Oxidation-reduction process
ENSG00000167770	OTUB1	OTU deubiquitinase, ubiquitin aldehyde binding 1	2.02	0.01970237	DNA repair
ENSG00000133112	TPT1	tumor protein, translationally-controlled 1	2.01	0	Calcium ion transport
ENSG00000126768	TIMM17B	translocase of inner mitochondrial membrane 17 homolog B (yeast)	1.99	0.02382261	Mitochondrial function
ENSG00000163479	SSR2	signal sequence receptor, beta (translocon-associated protein beta)	1.99	0.00000268	Translatin
ENSG00000084623	EIF3I	eukaryotic translation initiation factor 3 subunit I	1.98	0.00004869	Translation
ENSG00000105700	KXD1	KxDL motif containing 1	1.98	0.00349849	Vesicle mediated transport
ENSG00000110717	NDUFS8	NADH:ubiquinone oxidoreductase core subunit S8	1.98	0.00695935	Carbohydrate metabolism
ENSG00000184990	SIVA1	SIVA1, apoptosis-inducing factor	1.98	0.03640787	Apoptosis
ENSG00000225663	FAM195B	family with sequence similarity 195 member B	1.98	0.01036276	Cellular differentiation
ENSG00000103187	COTL1	coactosin-like F-actin binding protein 1	1.97	0.00939119	Uncharacterized
ENSG00000172270	BSG	basigin (Ok blood group)	1.95	0.00000159	Carbohydrate metabolism
ENSG00000172572	PDE3A	phosphodiesterase 3A	1.94	0.00000003	Energy metabolism
ENSG00000149923	PPP4C	protein phosphatase 4 catalytic subunit	1.93	0.00858809	DNA repair
ENSG00000105373	GLTSCR2	glioma tumor suppressor candidate region gene 2	1.92	0.01788232	RNA metabolism
ENSG00000128309	MPST	mercaptopyruvate sulfurtransferase	1.92	0.02464825	Cystein catabolism
ENSG00000130165	ELOF1	ELF1 homolog, elongation factor 1	1.92	0.03750893	Transcription
ENSG00000165283	STOML2	stomatin like 2	1.92	0.0036842	Calcium transport. Mitochondrial function
ENSG00000116649	SRM	spermidine synthase	1.91	0.02651626	Nitrogen metabolism
ENSG00000135930	EIF4E2	eukaryotic translation initiation factor 4E family member 2	1.9	0.02594661	Translation
ENSG00000125971	DYNLRB1	dynein, light chain, roadblock-type 1	1.89	0.00770092	Motor protein
ENSG00000143575	HAX1	HCLS1 associated protein X-1	1.89	0.00104179	Signalling
ENSG00000161016	RPL8	ribosomal protein L8	1.89	0.0000024	Translation
ENSG00000060138	YBX3	Y-box binding protein 3	1.88	0.00235393	Transcription
ENSG00000117691	NENF	neudesin neurotrophic factor	1.88	0.03199731	Neuronal function
ENSG00000184009	ACTG1	actin gamma 1	1.88	0	Cytoskeleton
ENSG00000204525	HLA-C	major histocompatibility complex, class I,	1.88	0.00263811	Immune response
ENSG00000087088	BAX	BCL2-associated X protein	1.85	0.02346751	Apoptosis
ENSG00000100348	TXN2	thioredoxin 2	1.85	0.01840015	Stress response
ENSG00000125991	ERGIC3	ERGIC and golgi 3	1.84	0.00980718	Vesicle mediated transport
ENSG00000150991	UBC	ubiquitin C	1.84	0.00000185	Signalling
ENSG00000160932	LY6E	lymphocyte antigen 6 complex, locus E	1.83	0.03750893	Immune response
ENSG00000123131	PRDX4	peroxiredoxin 4	1.82	0.01549798	Stress response
ENSG00000106628	POLD2	polymerase (DNA directed), delta 2, accessory subunit	1.81	0.01273685	Replication

ENSG00000118816	CCNI	cyclin I	1.81	0.00091177	Cell cycle
ENSG00000166165	CKB	creatine kinase, brain	1.81	0.00000131	Energy metabolism
ENSG00000203950	FAM127B	family with sequence similarity 127 member B	1.81	0.04873123	Uncharacteried
ENSG00000128272	ATF4	activating transcription factor 4	1.8	0.00000891	Transcription
ENSG00000083845	RPS5	ribosomal protein S5	1.79	0.00000126	Translation
ENSG00000142541	RPL13A	ribosomal protein L13a	1.78	0.00000008	Translation
ENSG00000146425	DYNLT1	dynein, light chain, Tctex-type 1	1.78	0.03195081	Motor protein
ENSG00000147677	EIF3H	eukaryotic translation initiation factor 3 subunit H	1.78	0.00200761	Translation
ENSG00000164054	SHISA5	shisa family member 5	1.78	0.03171697	Apoptosis
ENSG00000168298	HIST1H1E	histone cluster 1, H1e	1.78	0	Replication
ENSG00000198242	RPL23A	ribosomal protein L23a	1.78	0.00001225	Translation
ENSG00000213619	NDUFS3	NADH:ubiquinone oxidoreductase core subunit S3	1.78	0.03332656	Carbohydrate metabolism
ENSG00000155463	OXA1L	oxidase (cytochrome c) assembly 1-like	1.77	0.00980718	Mitochondrial function
ENSG000000159111		Mitochondrial ribosomal protein L10	1.77	0.03922492	Mitochondrial function
ENSG00000163931	TKT	transketolase	1.77	0.00096861	Carboyidrate metabolism
ENSG00000101220	C20orf27	chromosome 20 open reading frame 27	1.76	0.04873123	Uncharacterized
ENSG00000104897	SF3A2	splicing factor 3a subunit 2	1.76	0.03652086	RNA metabolism
ENSG00000233822	HIST1H2BN	histone cluster 1, H2bn	1.76	0.00091177	Replication
ENSG00000111481	COPZ1	coatomer protein complex subunit zeta 1 [	1.75	0.00700361	Vesicle mediated transport
ENSG00000125743	SNRPD2	small nuclear ribonucleoprotein D2 polypeptide	1.75	0.00010455	RNA metabolism
ENSG00000005022	SLC25A5	solute carrier family 25 (mitochondrial carrier; adenine nucleotide translocator), me	1.74	0.00000369	Mitochondrial function
ENSG00000087460	GNAS	GNAS complex locus	1.74	0.00000118	Signalling
ENSG00000143418	CERS2	ceramide synthase 2	1.73	0.0177841	Lipid metabolism
ENSG00000170889	RPS9	ribosomal protein S9	1.73	0.00000125	Translation
ENSG00000157020	SEC13	SEC13 homolog, nuclear pore and COPII coat complex component	1.72	0.04661418	Vesicle mediated transport
ENSG00000162517	PEF1	penta-EF-hand domain containing 1	1.72	0.02116997	Calcium ion transport
ENSG00000084207	GSTP1	glutathione S-transferase pi 1	1.71	0.00399616	Glutathion metabolism
ENSG00000167747	C19orf48	chromosome 19 open reading frame 48	1.71	0.00743596	Uncharacterized
ENSG00000104529	EEF1D	eukaryotic translation elongation factor 1 delta (guanine nucleotide exchange protei	1.7	0.00064714	Translation
ENSG00000172757	CFL1	cofilin 1 (non-muscle)	1.7	0.00006372	Cytoskeleton
ENSG00000177600	RPLP2	ribosomal protein, large, P2	1.69	0.0000404	Translation
ENSG00000198034	RPS4X	ribosomal protein S4, X-linked	1.69	0	Translation
ENSG00000274267	HIST1H3B	histone cluster 1, H3b	1.69	0.00000011	Replication
ENSG00000163046	ANKRD30E	ankyrin repeat domain 30B-like	1.68	0.00524849	Protein binding
ENSG00000167468	GPX4	glutathione peroxidase 4	1.68	0.00079564	Glutathion metabolism
ENSG00000100316	RPL3	ribosomal protein L3	1.67	0	Translation
ENSG00000135390	ATP5G2	ATP synthase, H+ transporting, mitochondrial Fo complex subunit C2 (subunit 9)	1.67	0.00000245	Mitochondrial function
ENSG00000105701	FKBP8	FK506 binding protein 8	1.66	0.03652086	Protein folding
ENSG00000115268	RPS15	ribosomal protein S15	1.66	0.00602621	Translation
ENSG00000111669	TPI1	triosephosphate isomerase 1	1.65	0.00109433	Carbohydrate metabolic process
ENSG00000147889	CDKN2A	cyclin-dependent kinase inhibitor 2A	1.65	0.04803903	Cell cycle
ENSG00000167996	FTH1	ferritin, heavy polypeptide 1	1.65	0.03435313	Ion transport
ENSG00000179271	GADD45GI	GADD45G interacting protein 1	1.65	0.03199731	Cell cycle
ENSG00000075624	ACTB	actin, beta	1.64	0.00000147	Cytoskeleton
ENSG00000108561	C1QBP	complement component 1, q subcomponent binding protein	1.63	0.00184143	RNA metabolism
ENSG00000167526	RPL13	ribosomal protein L13	1.63	0.00013687	Translation
ENSG00000179091	CYC1	cytochrome c-1	1.62	0.04104546	Mitochondrial function
ENSG00000142534	RPS11	ribosomal protein S11	1.61	0	Translation
ENSG00000166710	B2M	beta-2-microglobulin	1.61	0.04442378	Immune response
ENSG00000179218	CALR	calreticulin	1.61	0.00004648	Signalling
ENSG00000215021	PHB2	prohibitin 2	1.61	0.00579139	Transcription
ENSG00000169223	LMAN2	lectin, mannose-binding 2	1.61	0.02267609	
ENSG00000122705	CLTA	clathrin, light chain A	1.59	0.03888922	Vesicle mediated transport
ENSG00000136938	ANP32B	acidic nuclear phosphoprotein 32 family member B	1.58	0.01666639	Vesicle mediated transport
ENSG00000136942	RPL35	ribosomal protein L35	1.58	0.00000004	Translation
ENSG00000147403	RPL10	ribosomal protein L10	1.58	0.00114616	Translation
ENSG00000148303	RPL7A	ribosomal protein L7a	1.58	0.00000161	Translation
ENSG00000162244	RPL29	ribosomal protein L29	1.57	0.00089994	Translation
ENSG00000183010	PYCR1	pyrroline-5-carboxylate reductase 1	1.57	0.00096238	Aminoacid metabolism
ENSG00000187840	EIF4EBP1	eukaryotic translation initiation factor 4E binding protein 1	1.57	0.00824482	Translation
ENSG00000149100	EIF3M	eukaryotic translation initiation factor 3 subunit M	1.56	0.02158198	Translation
ENSG00000108298	RPL19	ribosomal protein L19	1.55	0	Translation
ENSG00000130770	ATPIF1	ATPase inhibitory factor 1	1.55	0.01962292	Energy metabolism
ENSG00000203814	HIST2H2BF	histone cluster 2, H2bf	1.55	0.0445242	Replication
ENSG00000274997	HIST1H2AH	histone cluster 1, H2ah	1.55	0.00002465	Replication
ENSG00000105202	FBL	fibrillarin	1.55	0.00005926	RNA metabolism
ENSG00000156508	EEF1A1	eukaryotic translation elongation factor 1 alpha 1	1.54	0.00194556	Translation
ENSG00000086598	TMED2	transmembrane p24 trafficking protein 2	1.53	0.00835398	Vesicle mediated transport
ENSG00000105438	KDELR1	KDEL endoplasmic reticulum protein retention receptor 1	1.53	0.04916751	Vesicle mediated transport
ENSG00000146701	MDH2	malate dehydrogenase 2 [	1.53	0.00779623	Carbohydrate metabolism
ENSG00000010256	UQCRC1	ubiquinol-cytochrome c reductase core protein I	1.52	0.01476154	Mitochondrial function
ENSG00000149273	RPS3	ribosomal protein S3	1.52	0	Translation
ENSG00000026025	VIM	vimentin	1.51	0.00175143	Motor protein

ENSG00000117592	PRDX6	peroxiredoxin 6	1.51	0.01215912	Oxidative stress
ENSG00000137818	RPLP1	ribosomal protein, large, P1	1.51	0.00243443	Translation
ENSG00000221983	UBA52	ubiquitin A-52 residue ribosomal protein fusion product 1	1.51	0.00733379	Signalling
ENSG00000278828	HIST1H3H	histone cluster 1, H3h	1.51	0.0030429	Replication
