# Supplementary material for: hCLE/RTRAF-HSPC117-DDX1-FAM98B: A New Cap-Binding Complex That Activates mRNA Translation
Source: Front Physiol. 2019 Feb 18;10:92. doi: 10.3389/fphys.2019.00092 (PMC6388641; doi:10.3389/fphys.2019.00092)
Supplement: Supplementary file 9 [file Data_Sheet_9.PDF]

Supp. Table S6. Predicted motifs found in 3' UTRs of hCLE-CBD bound RNAs.

Rate	3' UTR *de novo* motif results	P-value	% of Targets	% of Backgr.	Best Match/Details
1	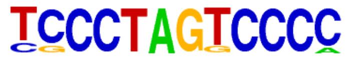	1e-32	4.42%	0.15%	PB0200.1_Zfp187_2/Jaspar(0.727)
2	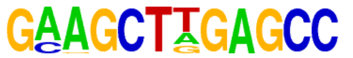	1e-29	4.56%	0.24%	ZNF415(Zf)/HEK293-ZNF415.GFP-ChIP-Seq(GSE58341)/Homer(0.803)
3	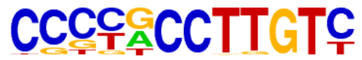	1e-29	5.25%	0.37%	SOX10/MA0442.1/Jaspar(0.684)
4	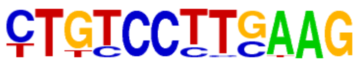	1e-26	4.97%	0.38%	Nr5a2(NR)/mES-Nr5a2-ChIP-Seq(GSE19019)/Homer(0.745)
5	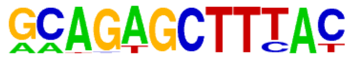	1e-25	4.56%	0.30%	DOF2(C2C2(Zn) Dof)/Zea mays/AthaMap(0.670)
6	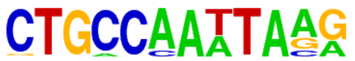	1e-25	4.56%	0.31%	NFIA/MA0670.1/Jaspar(0.754)
7	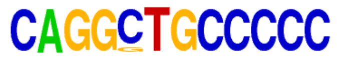	1e-25	4.14%	0.24%	shn/dmmpmm(Pollard)/fly(0.657)
8	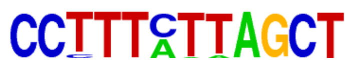	1e-25	3.45%	0.13%	PABPC1(?)/MEL-PABC1-CLIP-Seq (GSE69755)/Homer(0.650)
9	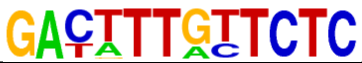	1e-24	4.42%	0.32%	PB0071.1_Sox4_1/Jaspar(0.650)
10	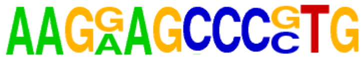	1e-22	4.56%	0.39%	MSN2(MacIsaac)/Yeast(0.655)
11	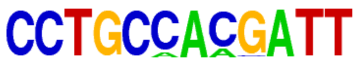	1e-22	3.31%	0.15%	RIM101/Literature(Harbison)/Yeast(0.645)
12	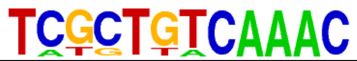	1e-20	3.18%	0.17%	Achi/dmmpmm(Noyes_hd)/fly(0.792)
13	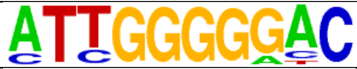	1e-19	6.08%	1.01%	PB0201.1_Zfp281_2/Jaspar(0.788)
14	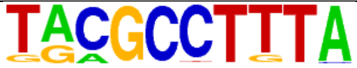	1e-19	4.14%	0.41%	PB0180.1_Sp4_2/Jaspar(0.696)
15	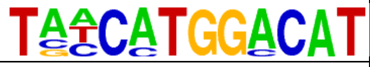	1e-18	2.62%	0.11%	LEC2/MA0581.1/Jaspar(0.617)
16	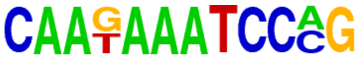	1e-17	2.07%	0.05%	Hoxc9/MA0485.1/Jaspar(0.679)
17	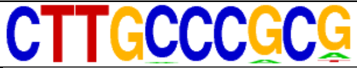	1e-17	4.01%	0.43%	SOK2(MacIsaac)/Yeast(0.729)
18	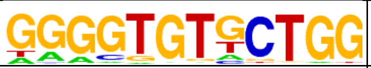	1e-17	5.94%	1.09%	AFT2/MA0270.1/Jaspar(0.698)
19	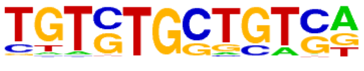	1e-15	4.97%	0.88%	Smad4(MAD)/ESC-SMAD4-ChIP-Seq (GSE29422)/Homer(0.709)
20	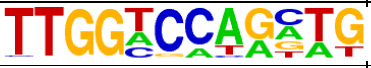	1e-14	4.14%	0.63%	HAP2/MA0313.1/Jaspar(0.742)
21	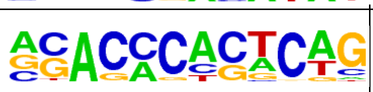	1e-14	2.76%	0.24%	Egr1(Zf)/K562-Egr1-ChIP-Seq(GSE32465)/Homer(0.747)
22	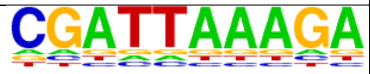	1e-13	2.21%	0.13%	CG34031/dmmpmm(Noyes_hd)/fly(0.746)
23	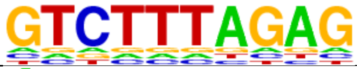	1e-12	2.35%	0.18%	RLM1(MacIsaac)/Yeast(0.683)
24	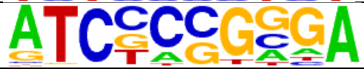	1e-12	3.18%	0.44%	OAF1/MA0348.1/Jaspar(0.734)

Supp. Table S6. Predicted motifs found in 3' UTRs of hCLE-CBD bound RNAs.

Table showing 3' UTR motifs detected in RNAs enriched in hCLE-CBD with a fold change >1.5, rated according to P-value signification. Second column shows a graphic representation of motive consensus according to nucleotide probability matrix. "% Targets" and "% Background" shows percentage of 3'UTR of our targets mRNAs or total 3'UTR population from ENSEMBL database, respectively. "Best Match/Detail" shows other mRNAs from different organisms that presents motifs similar to those selected. The platform used for this analysis was HOMER, by the application of the prediction algorithm "findMotifs" in default parameters.
